# Mechanisms of circular RNA circ_0066147 on pancreatic cancer progression

**DOI:** 10.1515/biol-2021-0047

**Published:** 2021-05-22

**Authors:** Jie Zhang, Zhang Zhang

**Affiliations:** Second Department of Tumor Intervention, The Second People’s Hospital of Wuhu, No. 259 Jiuhua Middle Road, Jinghu District 241000, Wuhu, Anhui, China

**Keywords:** pancreatic cancer, circ_0066147, miR-326, E2F2

## Abstract

**Background:**

The purpose of the study was to explore the precise parts of circ_0066147 (circular RNA [circRNA] scm-like with four mbt domains 1, circSFMBT1) in pancreatic cancer (PC) progression.

**Methods:**

Ribonuclease R assay was used to confirm the stability of circ_0066147. circ_0066147, miR-326 and E2F transcription factor 2 (E2F2) expression levels was detected by quantitative reverse-transcription polymerase chain reaction or Western blot. Cell proliferation, apoptosis, migration and invasion abilities were assessed by the 3-(4,5-dimethylthiazol-2-yl)-2,5-diphenyl-2*H*-tetrazolium bromide, flow cytometry, wound-healing and transwell assays, respectively. Targeted relationships among circ_0066147, miR-326 and E2F2 were verified by the dual-luciferase reporter or RNA pull-down assay.

**Results:**

circ_0066147 expression was upregulated in PC tissues and cells. circ_0066147 knockdown inhibited PC cell proliferation, migration, invasion and enhanced apoptosis *in vitro*, as well as weakened tumor growth *in vivo*. Mechanistically, circ_0066147 directly targeted miR-326 and circ_0066147 modulated E2F2 expression by miR-326. miR-326 mediated the regulation of circ_0066147 in PC cell behaviors *in vitro*. Furthermore, E2F2 was a functional target of miR-326 in modulating PC cell behaviors *in vitro*.

**Conclusion:**

circ_0066147 regulated PC malignant progression in part depending on the miR-326/E2F2 axis, illuminating circ_0066147 was a potential prognostic marker and therapeutic target for PC management.

## 1 Introduction

Pancreatic cancer (PC) is one of the most lethal malignancies worldwide [1]. Despite significant improvement in diagnosis and treatment, the 5-year survival rate of PC is under 6% [1,2]. The etiology of PC is complicated, and a better understanding of the molecular determinants of PC development will provide a unique opportunity to design novel therapeutic interventions [3].

Circular RNAs (circRNAs) are endogenous, continuous loop RNAs that exert important functions in normal development and disease [4]. Growing evidence has identified that some circRNAs function as posttranscriptional modulators of gene expression by targeting microRNAs (miRNAs) in various cancers, including PC [5,6]. For example, Hao et al. uncovered the oncogenic property of circ_0007534 in PC via targeting miR-892b and miR-625 [7]. Xu et al. demonstrated that circ_0030235 contributed to the carcinogenesis of PC by reducing the activity of miR-1294 and miR-1253 [8]. An et al. identified circ_0099999 as a strong tumor ogenic driver in PC which could directly repress miR-335-5p function [9]. As for circ_0066147 (also known as hsa_circ_103390, we confirmed that they were the same molecule by using the blast tool of circBase online database with the sequence of hsa_circ_103390 probe from the Gene Expression Omnibus [GEO] database), generated by the backsplicing of scm-like with four mbt domains 1 (SFMBT1) mRNA, it had been shown to be significantly overexpressed in pancreatic ductal adenocarcinoma (PDAC) using the GEO database [10,11]. Moreover, circ_0066147 was identified as an oncogenic driver in PC by enhancing cancer growth and metastasis [12]. Although it was previously reported that miR-330-5p/p21-activated kinase 1 (PAK1) axis was a downstream effector of circ_0066147 function [12], our understanding of its molecular basis is still limited.

miRNAs are small, endogenous noncoding RNAs, and dysregulation of miRNA have been implicated in the development of PC [13,14]. miR-326 has been identified as a promising biomarker for cancer diagnosis [15,16]. Moreover, miR-326 has been identified as a tumor suppressor in various cancers, such as gastric cancer, endometrial cancer and breast cancer [17,18,19]. The important involvement of miR-326 in PC carcinogenesis was predicted by a bioinformatics method [20]. Zhang et al. uncovered that miR-326, an underexpressed miRNA in PDAC, improved the survival of PC by repressing tumor cell proliferation and migration [21]. However, it is still unclear whether the oncogenic role of circ_0066147 in PC is mediated by miR-326.

Previous work showed that E2F transcription factor 2 (E2F2) was differently expressed in PC and was involved in the malignant progression of PC [22]. However, the precise roles of E2F2 in PC progression remain to be elucidated. When we used online prediction algorithms to help identify the molecular basis underlying circ_0066147-mediated promotion in PC, we found two putative binding sites between miR-326 and circ_0066147 or E2F2, implying a novel regulatory network in PC progression. For these reasons, we set to investigate whether the miR-326/E2F2 axis represents a downstream effector of circ_0066147 function. Here we showed that circ_0066147, an overexpressed circRNA in PC, contributed to PC progression by targeting the miR-326/E2F2 axis.

## 2 Materials and methods

### 2.1 Bioinformatics

The abnormally expressed circRNAs in PC tissues (*P* < 0.05, FC > 2 or FC < 2) were analyzed using the two public data sets (GSE79634 and GSE69362) from the GEO database (http://www.ncbi.nlm.nih.gov/geo). The overall survival of PC patients based on the level of E2F2 (patients were grouped according to the mean value of E2F2 level) was analyzed by the Cancer Genome Atlas (TCGA) database (http://gepia.cancer-pku.cn/detail.php). The directly interacted miRNAs of circ_0066147 were predicted by online databases circInteractome (https://circinteractome.nia.nih.gov/) and starBase (http://starbase.sysu.edu.cn/index.php/). Analysis of the molecular targets of miR-326 was performed using five search programs miRWalk (score ≥0.85, http://mirwalk.umm.uni-heidelberg.de/), TargetScan (http://www.targetscan.org/vert_71/), starBase, TarBase v7 (pred. score ≥0.7, http://www.microrna.gr/tarbase/) and miRDB (target score ≥50, http://mirdb.org/).

### 2.2 Clinical samples

The study population was a self-selected and informationally complete population of 45 patients (average age: 56.23) with primary PC at the Second People’s Hospital of Wuhu from September 2011 to December 2013. The clinicopathological features of these patients are summarized in Table 1. PC tissues and paired noncancerous tissues were collected from these volunteers and stored in liquid nitrogen. The follow-up information was obtained by telephone interview every 6 months until October 2018.

**Table 1:** Correlation between circ_0066147 expression and the clinicopathological features of 45 PC patients

Characteristic	All cases	circ_0066147 expression		*P* value
		High (*n* = 23)	Low (*n* = 22)	
**Gender**				0.641
male	25	12	13	
female	20	11	9	
**Age (years)**				0.458
<60	22	10	12	
≥60	23	13	10	
**Tumor size (cm)**				0.295
≥5	22	13	9	
<5	23	10	13	
**Lymph node metastasis**				0.011*
No	24	8	16	
Yes	21	15	6	
**Differentiation**				0.023*
Well/moderate	25	9	16	
Poor	20	14	6	

*P < 0.05 by chi-square test.


**Informed consent:** Informed consent has been obtained from all individuals included in this study.
**Ethical approval:** The research related to human use has been complied with all the relevant national regulations, institutional policies and in accordance with the tenets of the Helsinki Declaration and has been approved by the Ethics Committee of the Second People’s Hospital of Wuhu.

### 2.3 Cell lines

Human PC cell lines SW1990 (CRL-2172), PANC-1 (CRL-1469), Capan-2 (HTB-80), BxPC-3 (CRL-1687) and CFPAC-1 (CRL-1918) were purchased from American Type Culture Collection (ATCC, Manassas, VA, USA) and propagated using standard protocols provided by ATCC. The primary human pancreatic ductal epithelial (HPDE, isolated by our laboratory) cells were cultivated in serum-free keratinocyte medium plus bovine pituitary extract and epidermal growth factor (all from Gibco, Paisley, UK). All cells were routinely maintained at 37°C in 5% CO_2_.

### 2.4 Lentivirus transduction and transient transfection of cells

circ_0066147 overexpression plasmid (oe-circ_0066147) was constructed by inserting the full-length sequence of human circ_0066147 synthesized by Sangon Biotech (Shanghai, China) into a pCD5-ciR vector (Geneseed, Guangzhou, China) with EcoRI and BamHI restriction sites, and the matched negative plasmid (vector) was created in the same way with the scrambled control sequence. E2F2 overexpression plasmid (pcDNA-E2F2) was constructed by cloning human E2F2 (accession: NM_004091.4) into a pcDNA3.1 vector (Invitrogen, Paisley, UK) opened with NheI and XhoI sites, and a nonspecific pcDNA vector (pcDNA-NC) was used as the negative control. Human circ_0066147-specific siRNA (si-circ_0066147, 5′-GAGGTGCTTCCCTCCGGTTTT-3′) and nonspecific siRNA (si-NC, 5′-AAGACAUUGUGUGUCCGCCTT-3′), miR-326 mimic (5′-GACCUCCUUCCCGGGUCUCC-3′) and mimic control (miR-NC mimic, 5′-CGAUCGCAUCAGCAUCGAUUGC-3′), miR-326 inhibitor (anti-miR-326, 5′-GGAGACCCGGGAAGGAGGUC-3′) and inhibitor control (anti-miR-NC, 5′-CUAACGCAUGCACAGUCGUACG-3′) were purchased from Ribobio (Guangzhou, China). SW1990 and PANC-1 cells (1 × 10^4^ cells/well) were seeded in 24-well plates 12 h before transfection using Lipofectamine 2000 (Invitrogen) with 50 nM of siRNA, 25 nM of miRNA mimic or inhibitor and 100 ng of plasmid DNA. Further analyses of the transfected cells were carried out 48 h post-transfection.

Lentiviruses encoding circ_0066147-specific shRNA (sh-circ_0066147) and nontarget shRNA (sh-NC) were obtained from Geneseed. For stable cell lines, SW1990 and PANC-1 cells were infected with these virus particles in media containing 8 µg/mL polybrene (Yeasen, Shanghai, China). Twenty-four hours post-transduction, cells for selection were maintained in growth media in the presence of 2 µg/mL puromycin (Yeasen) more than 72 h.

### 2.5 Actinomycin D treatment

The assay was performed by incubating SW1990 and PANC-1 cells with Actinomycin D (Sigma-Aldrich, Toyko, Japan) in a final concentration of 2 mg/mL for the indicated time frame.

### 2.6 Preparation for RNA and protein

Total RNA and protein were extracted from tissue samples and cells using the AllPrep DNA/RNA Mini kit (Qiagen, Manchester, UK) per the accompanying directions. RNA was quantified by a NanoVue Plus spectrophotometer (GE Healthcare, Chicago, IL, USA). The concentration of protein extract was gauged based on the BCA method using an assay kit (Thermo Fisher Scientific, Waltham, MA, USA).

### 2.7 Ribonuclease R (RNase R) assay

RNase R assay was performed by adding 3 U RNase R (Geneseed) into per μg RNA and incubating at 37°C for 15 min.

### 2.8 Quantitative reverse transcription-polymerase chain reaction (qRT-PCR)

For circ_0066147 and E2F2 detection, cDNA was obtained according to the standard protocols using a SuperScript VILO cDNA Synthesis kit (Invitrogen) with random primers (Invitrogen); qRT-PCR analysis with SYBR Green (Qiagen) and designed primers (Table 1) was run in triplicate on a DNA Engine Opticon Monitor System (Bio-Rad, Glattbrugg, Switzerland). For miR-326 quantification, the miScript RT kit (Qiagen), miScript SYBR Green PCR kit (Qiagen) and designed primer (Table A1) were used. Data were analyzed by the comparative Ct (2^−ΔΔCt^) method [23] and presented as a corrected value by normalizing with the expression of the reference gene β-actin or U6.

### 2.9 Western blot

Proteins (50 μg) were electrophoresed on a 10% SDS polyacrylamide gel and then electroblotted onto a Clear Blot membrane-P (ATTO, Toyko, Japan). For primary antibody, antiproliferating cell nuclear antigen (anti-PCNA, 1:1,000 dilution, ab92552), anti-B-cell lymphoma-2 (anti-Bcl-2, 1:2,000 dilution, ab238041), anti-E2F2 (1:1,000 dilution, ab70731), anti-Cleaved-caspase3 (anti-C-caspase3, 1:1,000 dilution, ab214430), anti-Bcl-2-associated X (anti-Bax, 1:2,000 dilution, ab270742) and anti-glyceraldehyde-3-phosphate dehydrogenase (anti-GAPDH, 1:5,000 dilution, ab9484, all from Abcam, Cambridge, UK) antibodies were used. The secondary antibodies were horseradish peroxidase-conjugated anti-mouse and anti-rabbit IgG (ab97023 and ab97051, 1:5,000 dilution; Abcam). The bands were developed using the enhanced chemiluminescence (GE Healthcare) and analyzed with an LAS-4000 Image Analyzer (Fuji Photo Film, Toyko, Japan).

### 2.10 Cell proliferation and apoptosis assays

For proliferation analysis, transfected cells (2 × 10^3^ cells/well) were cultured in 96-well plates under standard protocols, and the 3-(4,5-dimethylthiazol-2-yl)-2,5-diphenyl-2*H*-tetrazolium bromide (MTT) assay kit (Abcam) was used per the accompanying guidance at every time point. The measured absorbance at 570 nm was proportional to the number of viable cells.

Cell apoptosis was evaluated by flow cytometry as previously described [24]. Briefly, transfected cells (1 × 10^6^ cells) were incubated with fluorescein isothiocyanate (FITC)-labeled Annexin-V (BD Biosciences, Heidelberg, Germany) and propidium iodide (PI; Invitrogen) in the dark for 15 min and analyzed within 30 min using an FACSCanto flow cytometer (BD Biosciences).

### 2.11 Cell migration and invasion assays

For migration analysis, a wound-healing assay was done by creating a vertical wound (0 h) through the cell monolayer (∼80% confluence) and incubating for 24 h at 37°C. The migratory rate was calculated as follows: migratory rate = (*A*_0_ − *A*_24_)/*A*_0_, where *A*_0_ represented the wound area of transfected cells at 0 h and *A*_24_ represented the wound area of transfected cells at 24 h.

For invasion analysis, a 24-transwell insert coated with Matrigel (BD Biosciences) was used. Transfected cells (1 × 10^5^ cells/well) in serum-free medium were seeded on the top of the insert membrane and 10% serum media was placed at the lower chamber. Following 24 h incubation, the invasive cells on the lower surface of the insert were stained with 0.2% crystal violet (Sigma-Aldrich). Five random fields of each sample were scored under a microscope at 100× magnification and the invasive cell population was examined by Image J software (NIH Image, Bethesda, MD, USA).

### 2.12 Dual-luciferase reporter assay

The segments of circ_0066147 and E2F2 3′UTR harboring the binding sites of miR-326 and the mutated sites in the target region were individually inserted into the psiCHECK-2 reporter vector (Promega, Madison, WI, USA). SW1990 and PANC-1 cells (1 × 10^4^ cells) were cotransfected with 250 ng of each luciferase reporter construct, 25 ng of phRL-SV40 control vector (Promega) and 25 nM of miR-326 mimic or mimic control using Lipofectamine 2000 (Invitrogen). Luciferase activity was detected using the Dual Luciferase Assay System (Promega).

### 2.13 RNA pull-down assay

Biotin-labeled miR-326 mimic (Bio-miR-326-WT), matched mutant in the target region (Bio-miR-326-MUT) and negative control (Bio-miR-NC) were obtained from Ribobio. Cell lysates were incubated with these probes at 4°C for 4 h before adding the streptavidin beads (Sigma-Aldrich) for 2 h. Beads were harvested and the total RNA bound to beads was extracted for circ_0066147 analysis.

### 2.14 Animal studies

SW1990 and PANC-1 cells transfected with sh-circ_0066147 or sh-NC were subcutaneously inoculated into the male BALB/c nude mice aged 6–8 weeks (*n* = 6 each group; Vital River Laboratory, Beijing, China). Tumor volume was periodically monitored by a digital caliper and determined using the formula: (length × width^2^) × 0.5. At end points, the mice were sacrificed and tumor masses were excised for weight and gene expression analyses.


**Ethical approval:** The research related to animal use has been complied with all the relevant national regulations and institutional policies for the care and use of animals and were approved by the Animal Care and Use Ethics Committee of the Second People’s Hospital of Wuhu.

### 2.15 Statistical analysis

All assays were repeated twice and done in triplicates each time. Results were presented as the mean ± standard deviation. A Student’s *t* test was used for the comparison between two groups, and analysis of variance with Tukey’s *post hoc* test was applied for analysis of three or more groups. The difference in gene expression in tissue samples was determined by the Mann–Whitney *U* test. Correlation between gene expression levels in PC tissues was analyzed using the Spearman correlation test. For survival analysis, the Kaplan–Meier method and log-rank test were used. Correlation between circ_0066147 expression and the clinicopathological features of these patients was evaluated by chi-square test. *P* < 0.05 was considered as a significant difference.

## 3 Results

### 3.1 circ_0066147 was overexpressed in PC tissues and cell lines

**Figure 1:**
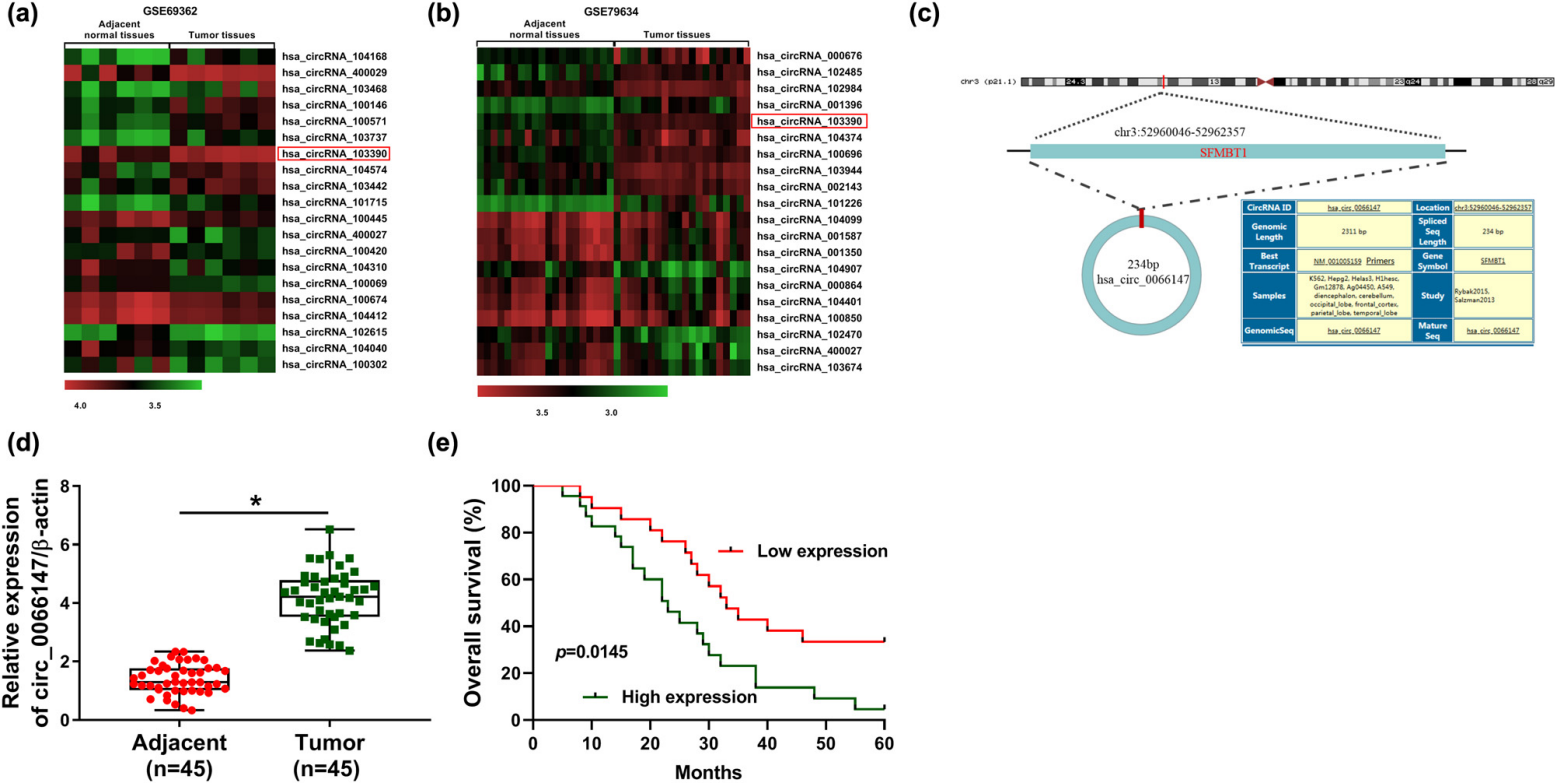
circ_0066147 level was upregulated in PC tissues. (a and b) The heat maps of abnormally expressed circRNAs in PC tissues from the GEO database. (c) Schematic of the covalently closed loop structure of circ_0066147. (d) Relative circ_0066147 level by qRT-PCR in 45 pairs of PC tissues and matched noncancerous tissues, with β-actin as the reference gene. (e) Survival analyses of these PC patients divided into a low circ_0066147 level group (*n* = 21) and a high circ_0066147 level group (*n* = 24), according to the mean value of circ_0066147 expression. *P < 0.05.

To identify the abnormally expressed circRNAs in PC, we analyzed two public data sets (GSE79634 and GSE69362) from the GEO database. The microarray data showed that many circRNAs were differentially expressed in PC tissues compared with matched normal controls, and one of them was circ_0066147 (also known as hsa_circ_103390) (Figure 1a and b). As shown in Figure 1c, circ_0066147 was 234 bp long that was produced by backspliced exons of SFMBT1 mRNA. Then qRT-PCR was used to assess the expression of circ_0066147 in PC tissues. circ_0066147 expression was upregulated in PC tissues compared with the adjacent noncancerous tissues (*P* < 0.0001; Figure 1d). To evaluate the clinical relevance of circ_0066147 expression, survival analysis was performed. Interestingly, we found that these patients with low circ_0066147 levels had a longer survival time than those with high circ_0066147 levels (*P* = 0.0145; Figure 1e). Additionally, circ_0066147 expression was associated with lymph node metastasis and differentiation of tumors (Table 1).

**Figure 2:**
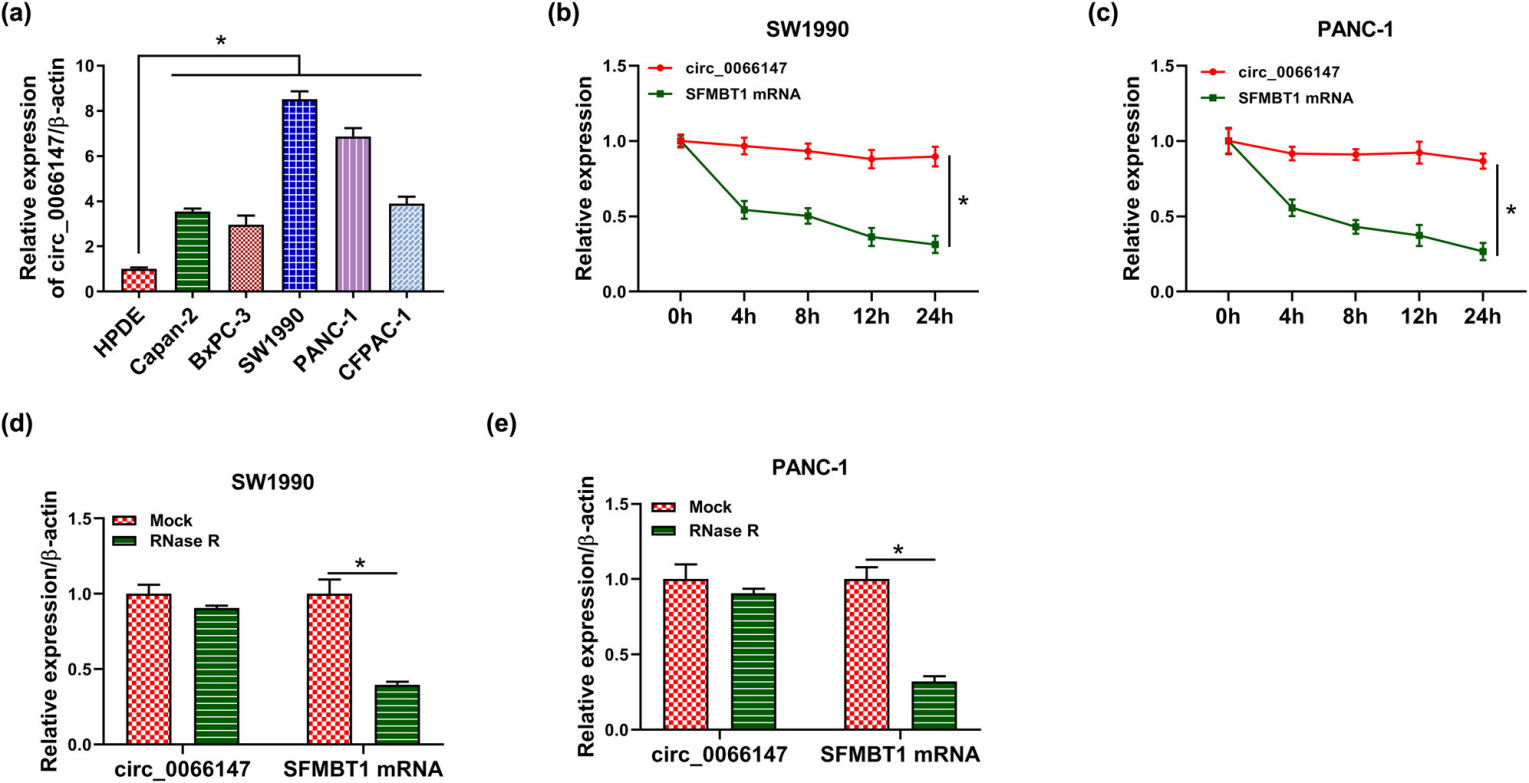
circ_0066147 was overexpressed in PC cells. (a) Relative circ_0066147 level by qRT-PCR in HPDE, Capan-2, BxPC-3, SW1990, PANC-1 and CFPAC-1 cell lines, with β-actin as the reference gene. (b and c) Expression level of circ_0066147 and SFMBT1 mRNA was detected in Actinomycin D-treated SW1990 and PANC-1 cells. β-actin served as the reference gene. (d and e) RNase R assays in SW1990 and PANC-1 cells. β-actin served as the reference gene. *P < 0.05.

Consistent with PC tissues, circ_0066147 was overexpressed in PC cells compared with HPDE cells (*P* < 0.0001; Figure 2a). To confirm the stability of circ_0066147, SW1990 and PANC-1 cells were treated with Actinomycin D, and RNase R assay was performed in both cell lines. As expected, Actinomycin D treatment led to a reduction in the level of SFMBT1 linear mRNA (*P* < 0.0001), while circ_0066147 level was not reduced (Figure 2b and c). RNase R assays showed that circ_0066147, rather than SFMBT1 linear mRNA (*P* < 0001), was resistant to RNase R (Figure 2d and e).

### 3.2 Knockdown of circ_0066147 suppressed cell proliferation, migration, invasion and promoted apoptosis *in vitro*

**Figure 3:**
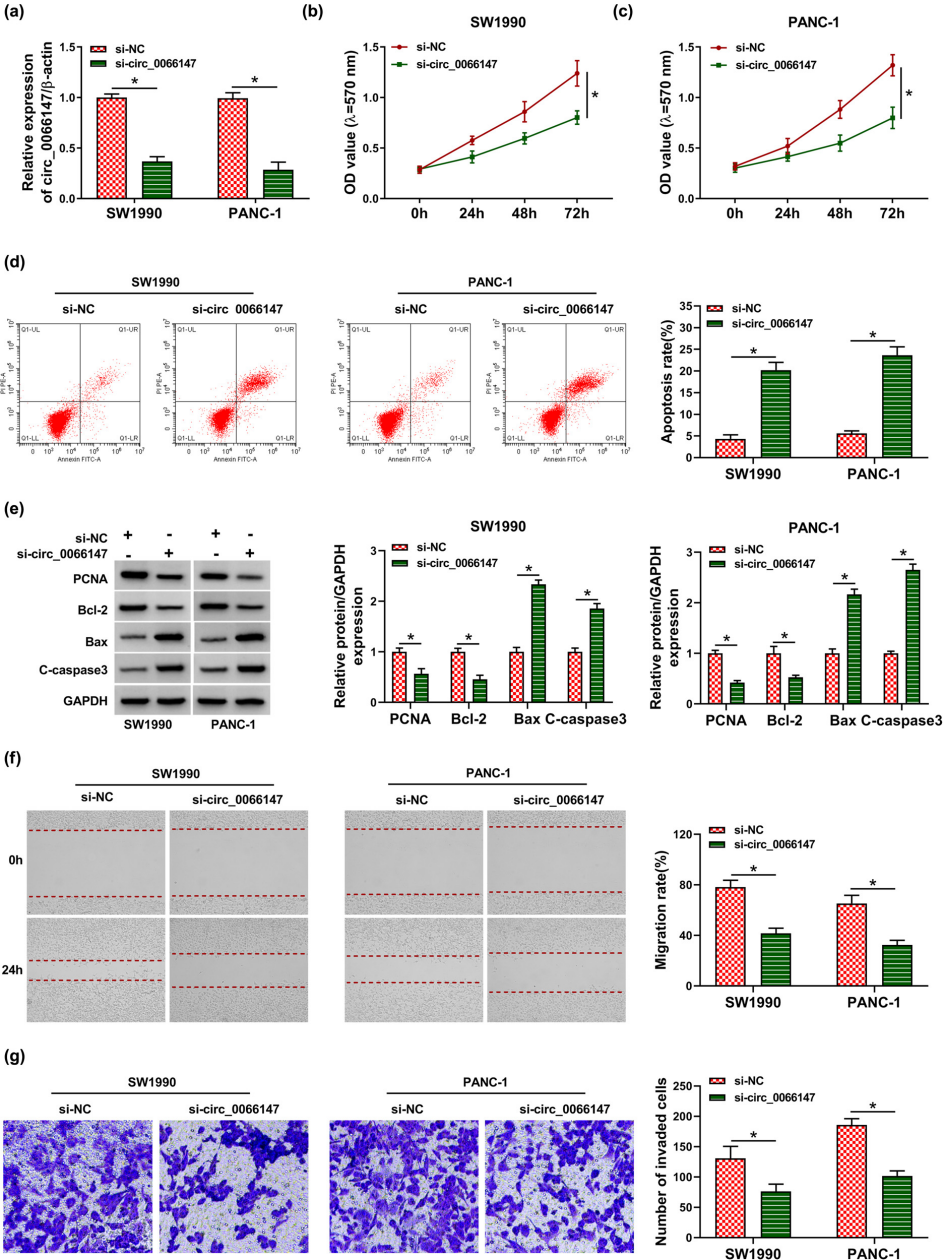
circ_0066147 silencing regulated cell proliferation, migration, invasion and apoptosis *in vitro*. SW1990 and PANC-1 cells were transfected with si-NC or si-circ_0066147. (a) qRT-PCR for circ_0066147 expression in transfected cells. β-Actin served as the reference gene. (b and c) MTT assay for cell proliferation. (d) Flow cytometry for cell apoptosis. (e) Western blot for PCNA, Bcl-2, Bax and C-caspase3 levels. GAPDH served as the reference gene. (f) Wound-healing assay for cell migration. (g) Transwell assay for cell invasion. *P < 0.05.

To elucidate the functional roles of circ_0066147 in PC progression, we performed “phenocopy” silencing in both SW1990 and PANC-1 cell lines with circ_0066147-siRNA (si-circ_0066147). Transient transfection of si-circ_0066147, but not the si-NC control, downregulated the expression of circ_0066147 in both cell lines (*P* < 0.0001; Figure 3a). By contrast, the silencing of circ_0066147 repressed cell proliferation (*P* < 0.0001; Figure 3b and c) and enhanced cell apoptosis (*P* < 0.0001; Figure 3d). Moreover, circ_0066147 knockdown decreased the levels of proliferation marker PCNA and antiapoptotic protein Bcl-2, while increased proapoptotic proteins Bax and C-caspase3 expression in two PC cell lines (*P* < 0.0001; Figure 3e), supporting the impact of circ_0066147 knockdown on cell proliferation and apoptosis. Furthermore, the knockdown of circ_0066147 repressed cell migration (*P* < 0.0001; Figure 3f) and invasion (*P* = 0.0022 or *P* = 0.0001; Figure 3g) in SW1990 and PANC-1 cells.

### 3.3 Circ_0066147 directly targeted miR-326

**Figure 4:**
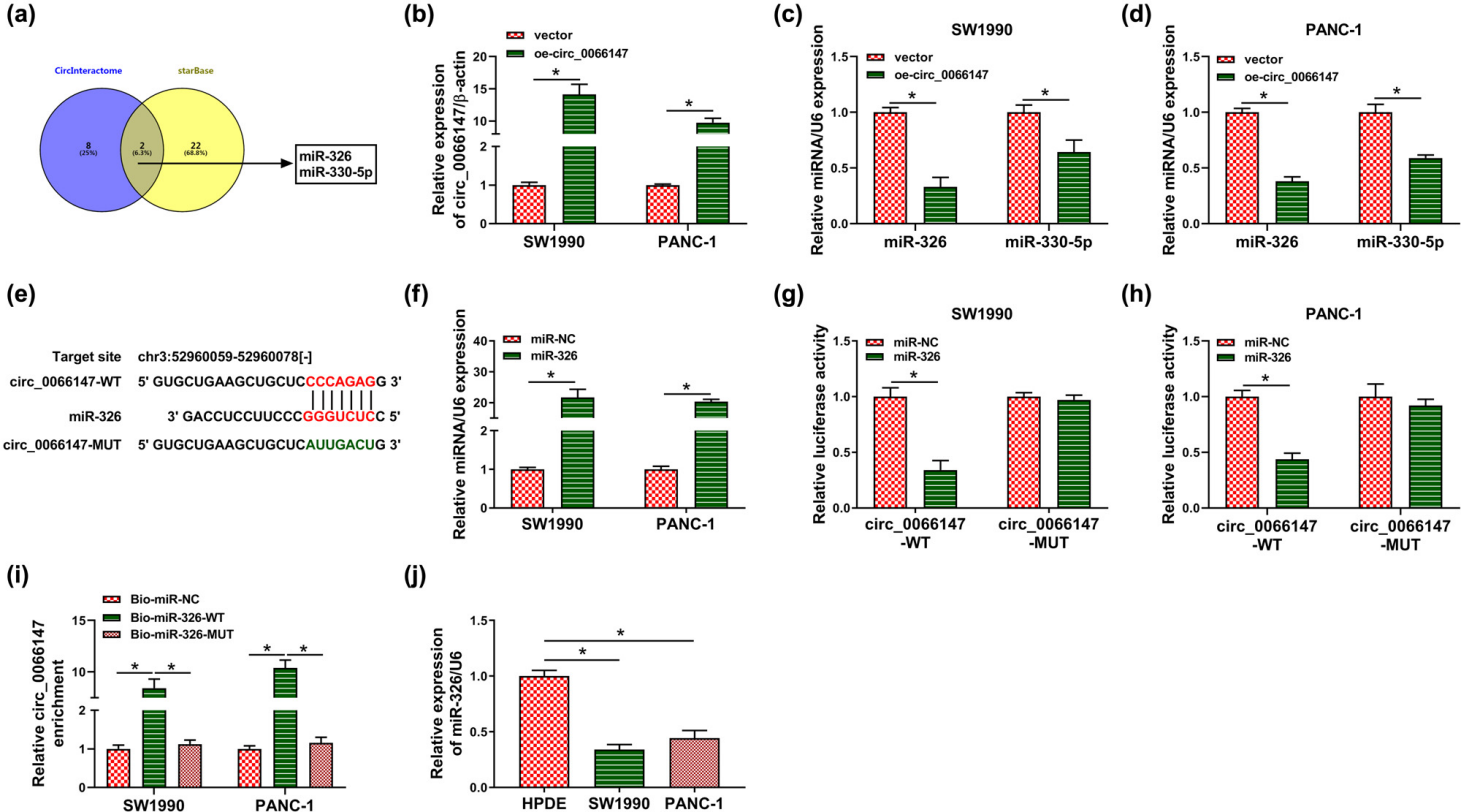
circ_0066147 in PC cells targeted miR-326 by directly binding to miR-326. (a) Venn diagram of the targeted miRNAs of circ_0066147 predicted by circInteractome and starBase online databases. (b–d) Relative levels of circ_0066147, miR-326 and miR-330-5p by qRT-PCR in SW1990 and PANC-1 cells transfected with vector negative plasmid or circ_0066147 overexpression plasmid (oe-circ_0066147). β-Actin or U6 served as the reference gene. (e) Schematic of the miR-326-binding region within circ_0066147 and mutated the target sequence. (f) Relative miR-326 expression in SW1990 and PANC-1 cells transfected with miR-NC mimic or miR-326 mimic. U6 served as the reference gene. (g and h) Dual-luciferase reporter assays in SW1990 and PANC-1 cells. (i) RNA pull-down assays in SW1990 and PANC-1 cells. β-Actin served as the reference gene. (j) Relative miR-326 expression by qRT-PCR in HPDE, SW1990 and PANC-1 cells. U6 served as the reference gene. *P < 0.05.

**Figure A1:**
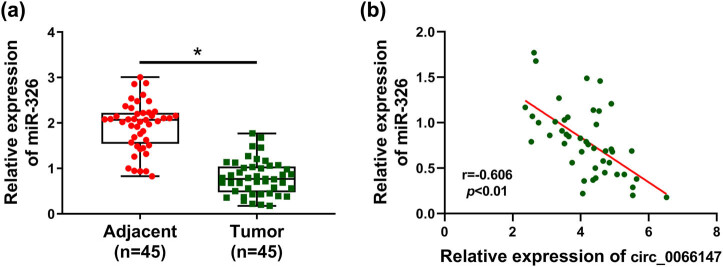
The level of miR-326 was reduced in PC tissues. (a) Relative miR-326 expression by qRT-PCR in 45 pairs of PC tissues and matched noncancerous tissues. U6 served as the reference gene. (b) Correlation between circ_0066147 and miR-326 expression levels in PC tissues using the Spearman correlation test. *P < 0.05.

To determine the mechanism of circ_0066147 on PC progression, we used the online algorithms (circInteractome and starBase) to identify its targeted miRNAs. The results showed that miR-326 and miR-330-5p were two putative targeted miRNAs of circ_0066147 (Figure 4a). We then observed whether circ_0066147 influenced endogenous expression of miR-326 and miR-330-5p in both SW1990 and PANC-1 cell lines. The transfection efficiency of circ_0066147 overexpression plasmid (oe-circ_0066147) was validated by qRT-PCR (*P* < 0.0001; Figure 4b). Importantly, the enforced expression of circ_0066147 led to a downregulated expression in both cell lines of miR-326 and miR-330-5p (*P* < 0.0001 or *P* = 0.0011; Figure 4c and d). To confirm whether circ_0066147 directly interacted with miR-326, the circ_0066147 segment encompassing the miR-326-binding region was cloned into a luciferase plasmid to mutate the target sequence and then was tested for luciferase activity (Figure 4e). The transfection efficiency of miR-326 mimic in two PC cell lines was evaluated by qRT-PCR (*P* < 0.0001; Figure 4f). When we performed dual-luciferase reporter assays, the cotransfection of circ_0066147 luciferase reporter (circ_0066147-WT) and miR-326 mimic into two PC cell lines produced lower luciferase activity than cells cotransfected with miR-NC control, but the mutation of the seed region (circ_0066147-MUT) abrogated the repression of miR-326 (Figure 4g and h). RNA pull-down assays revealed that the incubation with biotin-labeled miR-326 mimic (Bio-miR-326-WT) caused an increase in circ_0066147 enrichment level (*P* < 0.0001), and the effect was abolished by the mutant of the seed region (Bio-miR-326-MUT; Figure 4i). Additionally, qRT-PCR showed the downregulation of miR-326 expression in PC tissues and cells (Figures 4j and A1a). Interestingly, a strong inverse correlation between circ_0066147 and miR-326 expression levels was discovered in PC tissues (Figure A1b).

### 3.4 miR-326 was a functional mediator of circ_0066147 in regulating PC cell proliferation, migration, invasion and apoptosis *in vitro*

**Figure 5:**
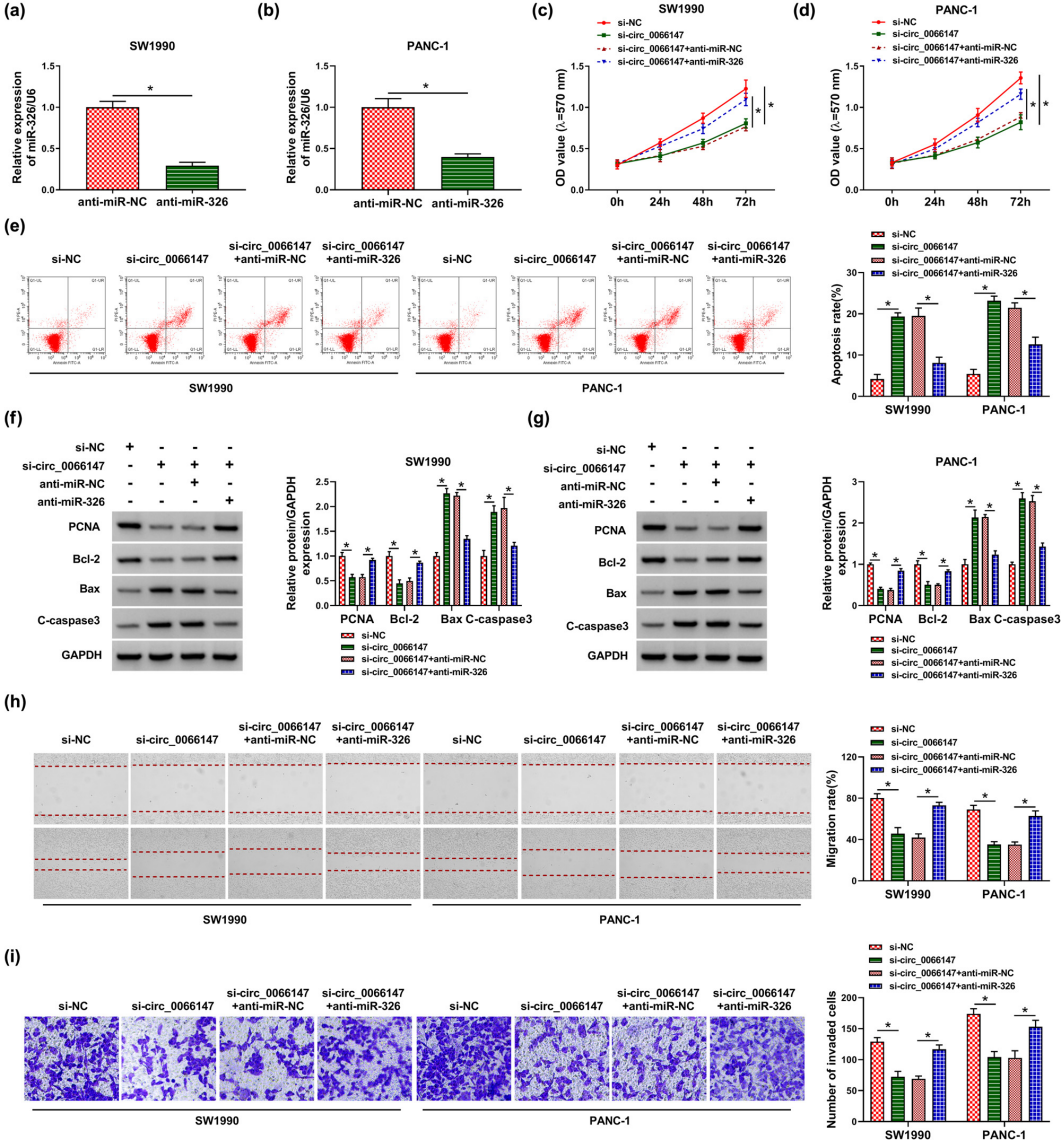
circ_0066147 regulated PC cell proliferation, migration, invasion and apoptosis *in vitro* by miR-326. (a and b) The level of miR-326 by qRT-PCR in SW1990 and PANC-1 cells transfected with anti-miR-NC or anti-miR-326. U6 served as the reference gene. SW1990 and PANC-1 cells were transfected with si-NC, si-circ_0066147, si-circ_0066147 + anti-miR-NC or si-circ_0066147 + anti-miR-326, followed by the detection of cell proliferation by MTT assay (c and d), cell apoptosis by flow cytometry (e), PCNA, Bcl-2, Bax and C-caspase3 levels by Western blot (f and g), cell migration by wound-healing assay (h), cell invasion by transwell assay (i). GAPDH served as the reference gene. *P < 0.05.

To test whether miR-326 was involved in the regulation of circ_0066147 on PC progression, the expression of miR-326 was blocked in circ_0066147-silencing PC cells. By contrast, the transfection of anti-miR-326 inhibited miR-326 expression in SW1990 and PANC-1 cells (*P* = 0.0001 or *P* = 0.0007; Figure 5a and b). Further analyses revealed that the downregulation of miR-326 abolished circ_0066147 knockdown-mediated antiproliferation, proapoptosis (Figure 5c–g), antimigration (Figure 5h) and anti-invasion (Figure 5i) effects in the two PC cell lines.

### 3.5 circ_0066147 modulated E2F2 expression through miR-326

**Figure 6:**
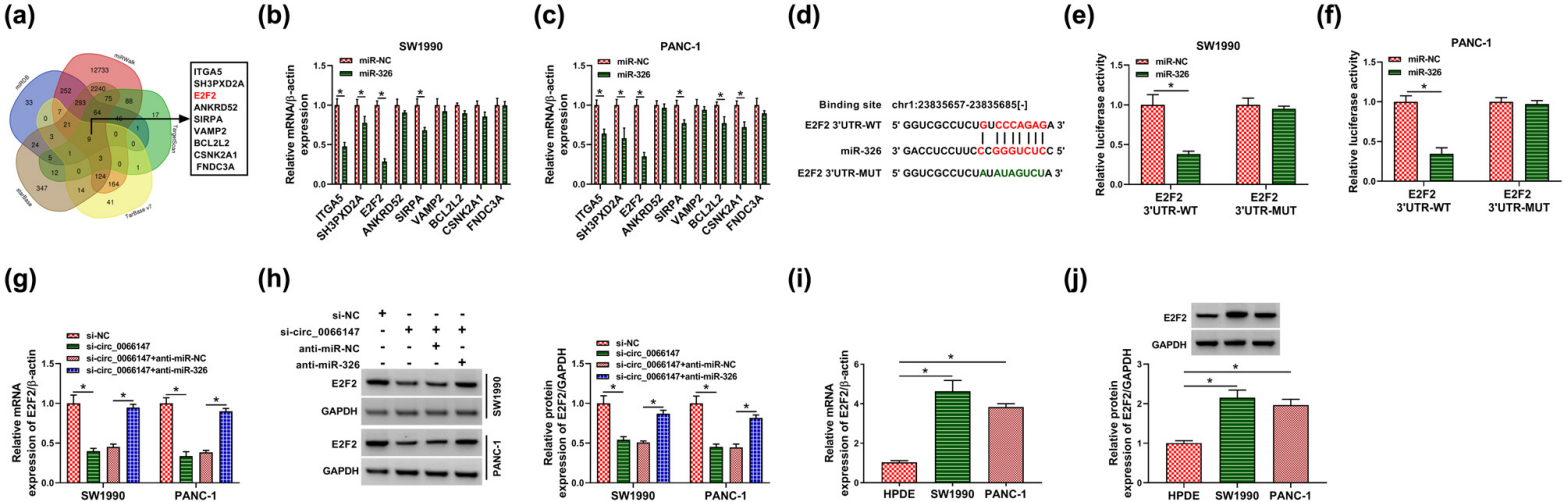
circ_0066147-mediated E2F2 expression via targeting miR-326. (a) Venn diagram of the putative targets of miR-326 predicted by miRWalk, TargetScan, starBase, TarBase v7 and miRDB search programs. (b and c) The expression levels of nine targets of miR-326 by qRT-PCR in SW1990 and PANC-1 cells transfected with miR-NC mimic or miR-326 mimic. β-Actin served as the reference gene. (d) Schematic of the putative miR-326-binding sites within E2F2 3′UTR identified by starBase database and the mutant of the seed sequence. (e and f) Dual-luciferase reporter assays in SW1990 and PANC-1 cells. (g and h) Relative E2F2 mRNA and protein levels in the cells transfected with si-NC, si-circ_0066147, si-circ_0066147 + anti-miR-NC or si-circ_0066147 + anti-miR-326. β-Actin or GAPDH served as the reference gene. (i and j) Relative E2F2 mRNA and protein levels in HPDE, SW1990 and PANC-1 cells. β-Actin or GAPDH served as the reference gene. *P < 0.05.

To identify downstream targets of miR-326, mRNA target-predicting algorithms (miRWalk, TargetScan, starBase, TarBase v7 and miRDB) were used. Interestingly, Venn diagram showed nine target conjuncts predicted by five algorithms (Figure 6a). We then determined whether miR-326 inhibited the expression of these target genes in SW1990 and PANC-1 cells. By contrast, miR-326 overexpression reduced the expression of several genes in both cell lines, and E2F2 was the most significantly downregulated target (*P* < 0.0001; Figure 6b and c). As shown in Figure 6d, eight putative complementary sites are observed for miR-326 within E2F2 3′UTR. To validate this, we performed dual-luciferase reporter assays using E2F2 3′UTR wild-type and mutant-type reports (E2F2 3′UTR-WT and E2F2 3′UTR-MUT). E2F2 3′UTR-WT and miR-326 overexpression caused a reduction in luciferase activity (*P* < 0.0001; Figure 6e and f). When the target sites were mutated, no reduction in luciferase was observed with miR-326 overexpression (Figure 6e and f). Altogether these data indicated that E2F2 was a direct target of miR-326 in PC cells.

**Figure A2:**
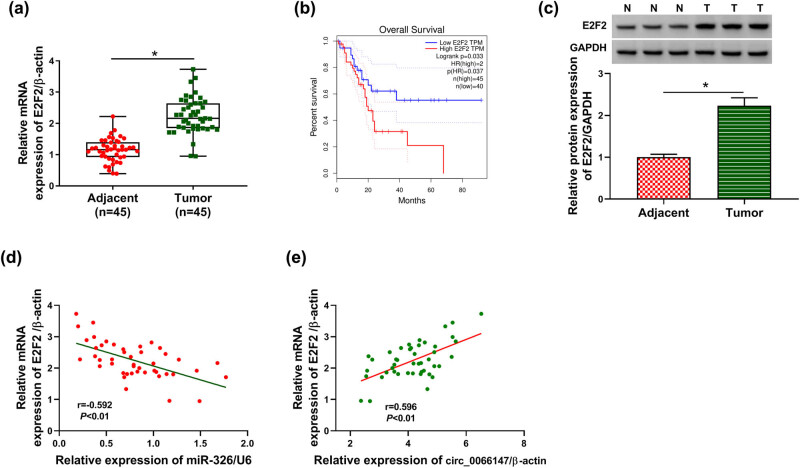
E2F2 was overexpressed in PC tissues. (a) E2F2 mRNA level by qRT-PCR in 45 pairs of PC tissues and matched noncancerous tissues. β-Actin served as the reference gene. (b) Survival analysis of PC patients based on the expression of E2F2 from the TCGA database. (c) E2F2 protein level by Western blot in three pairs of PC tissues and matched noncancerous tissues. GAPDH served as the reference gene. (d and e) Correlations among the E2F2, miR-326 and circ_0066147 levels in PC tissues using the Spearman correlation test. *P < 0.05.

Then whether circ_0066147 modulated E2F2 expression in PC cells was revealed. As expected, circ_0066147 silencing decreased the expression of E2F2 at both mRNA and protein levels (*P* < 0.0001), which was reversed by anti-miR-326 transfection (*P* < 0.0001; Figure 6g and h). E2F2 mRNA level increased in PC tissues compared with the negative controls (Figure A2a). Additionally, the TCGA database showed that PC patients with low E2F2 levels had a longer survival time than those with high E2F2 levels (Figure A2b). Moreover, E2F2 mRNA and protein levels were overexpressed in PC tissues and cells (Figures 6i–j and A2c). More interestingly, in PC tissues, E2F2 mRNA level was inversely correlated with miR-326 expression and positively correlated with circ_0066147 level (Figure A2d and e).

### 3.6 Enforced expression of miR-326 suppressed PC cell proliferation, migration, invasion and promoted apoptosis *in vitro* by downregulating E2F2

**Figure 7:**
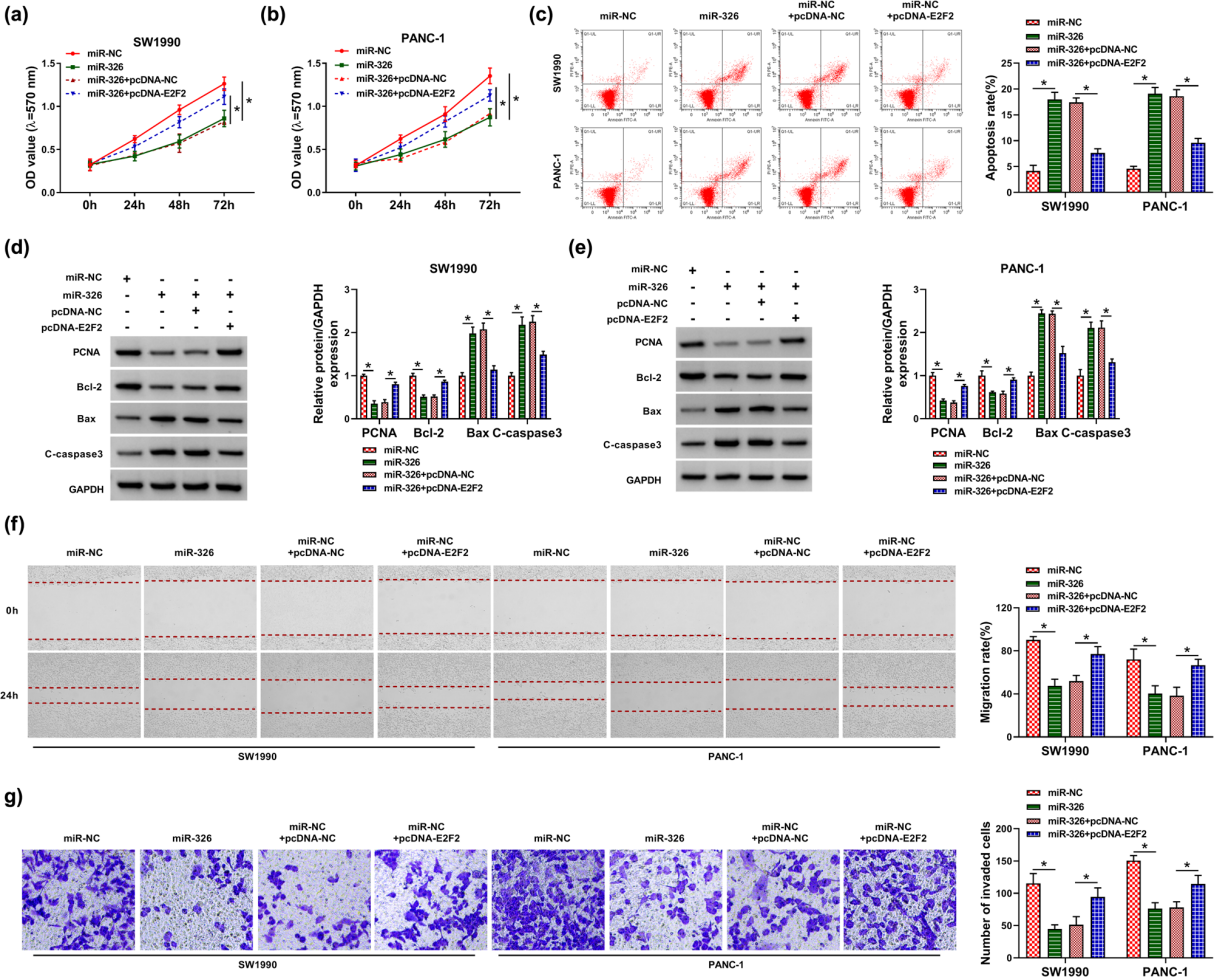
miR-326-modulated PC cell proliferation, migration, invasion and apoptosis *in vitro* by targeting E2F2. SW1990 and PANC-1 cells were transfected with miR-NC mimic, miR-326 mimic, miR-326 mimic + pcDNA-NC or miR-326 mimic + pcDNA-E2F2. (a and b) Cell proliferation by MTT assay. (c) Cell apoptosis by flow cytometry. (d and e) The levels of PCNA, Bcl-2, Bax and C-caspase3 by Western blot. GAPDH served as the reference gene. (f) Cell migration by wound-healing assay. (g) Cell invasion by transwell assay. *P < 0.05.

To determine whether E2F2 was a functional target of miR-326 in regulating PC progression, we transfected miR-326 mimic alone or together with E2F2 overexpression plasmid (pcDNA-E2F2). By contrast, enforced expression of miR-326 suppressed cell proliferation and enhanced cell apoptosis (Figure 7a–e). Moreover, miR-326 overexpression restrained cell migration (Figure 7f) and invasion (Figure 7g) in both cell lines. Furthermore, these effects of miR-326 overexpression were abrogated by the cotransfection of E2F2 overexpression plasmid (*P* < 0.0001; Figure 7a–g).

### 3.7 Knockdown of circ_0066147 weakened tumor growth *in vivo*

**Figure 8:**
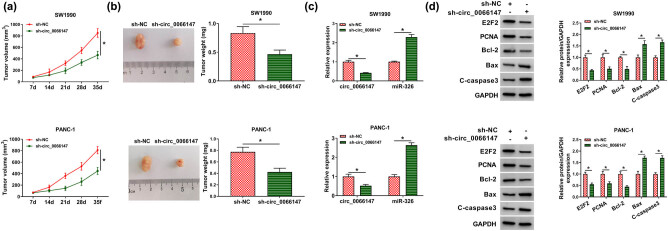
circ_0066147 silencing attenuated tumor growth *in vivo*. SW1990 and PANC-1 cells stably infected with sh-circ_0066147 or sh-NC were individually implanted into the nude mice (*n* = 6 each group). In 35 days postimplantation, mice were euthanized and tumor tissues were excised. (a) Growth curves of the xenograft tumors. (b) Representative images and the average weight of the excised tumors. circ_0066147 and miR-326 expression levels by qRT-PCR (c) and the levels of E2F2, PCNA, Bcl-2, Bax and C-caspase3 by Western blot (d) in the xenograft tissues. β-Actin, U6 or GAPDH served as the reference gene. *P < 0.05.

To unveil whether circ_0066147 could influence tumor growth *in vivo*, circ_0066147 level was blocked in SW1990 and PANC-1 cells and then sh-circ_0066147-infected or sh-NC-transduced PC cells were implanted into the nude mice. After the infection with sh-circ_0066147, the tumor growth was attenuated (*P* < 0.0001; Figure 8a and b). Moreover, circ_0066147 and E2F2 levels were downregulated and miR-326 expression was upregulated in sh-circ_0066147-transduced tumors (*P* < 0.0001; Figure 8c and d). Additionally, in line with the PC cells *in vitro*, circ_0066147 knockdown led to a distinct decrease in the levels of PCNA and Bcl-2, but an increase in Bax and C-caspase3 in the tumor tissues derived from sh-circ_0066147-transduced PC cells (*P* < 0.0001; Figure 8d).

## 4 Discussion

circRNAs have been implicated in human carcinogenesis [25]. Moreover, circRNA-miRNA-mRNA regulatory networks have been widely identified in cancer biology [26,27]. Here our results demonstrated that circ_0066147 was overexpressed in PC tissues and cells, which could contribute to PC progression *in vitro* and *in vivo*. Furthermore, we provided a molecular explanation for the oncogenic role of circ_0066147 in PC. In this study, we first revealed that the overexpression of circ_0066147 predicted poor prognosis of PC patients, suggesting circ_0066147 might be a potential marker for PC prognosis. As previously reported for other circRNAs [28,29], circ_0066147 was stable and resistant to RNase R because of the absence of free 3′ or 5′ ends [30].

miR-326 has been established as a promising biomarker for cancer diagnosis [15,16] and tumor inhibitor in various cancers [17,18,19]. miR-326 was also reported to be involved in PC development [21]. Our data first showed that circ_0066147 directly targeted miR-326. Furthermore, we pointed to the regulation of circ_0066147 in PC progression through miR-326. Similarly, Tang et al. uncovered that circ_0000515 contributed to the tumorigenesis of cervical cancer by inhibiting miR-326 activity [31]. Yu et al. highlighted that circ_0003998 worked as a tumor driver in non-small cell lung carcinoma by directly binding to miR-326 [32].

Several previous studies had demonstrated the conflicting roles of E2F2 in human carcinogenesis [33,34,35,36]. These contradictory findings might in part be due to the different types of tumors in these reports, where E2F2 functioned as a tumor suppressor in Myc-induced cancers [35,36] and exerted a strong oncogenic effect in lung cancer and glioblastoma cells [33,34]. In this study, it was first showed that E2F2 was a functionally important target of miR-326 in regulating PC progression *in vitro*. Previous work also reported several other miRNAs, such as miR-936 and let-7a, served as antitumor factors by targeting E2F2 [37,38]. More interestingly, we first identified circ_0066147 as a posttranscriptional regulator of E2F2 expression through miR-326. A recent document reported that circ_0066147 enhanced the development of PC by miR-330-5p-dependent regulation of PAK1 [12]. With these findings, we speculated that there might be other miRNA/mRNA networks that remain to be identified in the regulation of circ_0066147. Additionally, E2F2 can regulate the activation of PI3K/AKT and NF-kappaB signaling pathways [39,40], which play crucial roles in PC progression [41,42]. A future challenge will be to elucidate whether these pathways are involved in circ_0066147-mediated PC progression.

Collectively, the current study demonstrated that silencing of circ_0066147 suppressed PC malignant progression depending on the miR-326/E2F2 axis. Our findings highlighted circ_0066147 as a potential prognostic marker and therapeutic target for PC management.

## Appendix

**Table A1:** Sequences of qRT-PCR primers

Sequence (5′–3′)		
circ_0066147	Forward	TGACTGGGCTGACTACCTCA
	Reverse	CTCCACTGCACAGGGAAGAT
SFMBT1 linear mRNA	Forward	TGACTTGCGTCCTGAGAACC
	Reverse	AAAGTCCTGGCTTGGGTAGC
E2F2	Forward	AGACTCAAGGACTAGAGAGCG
	Reverse	TAGAGATCGCCGCTTGGAGA
miR-326	Forward	GCCGAGCCTTCCCGGGTCT
	Reverse	CAGTGCGTGTCGTGGAGT
U6	Forward	CTCGCTTCGGCAGCACA
	Reverse	AACGCTTCACGAATTTGCGT
β-Actin	Forward	CTCGCCTTTGCCGATCC
	Reverse	GGGGTACTTCAGGGTGAGGA
